# Masking for COVID-19 and other respiratory viral infections: implications of the available evidence

**DOI:** 10.1017/ash.2024.67

**Published:** 2024-05-03

**Authors:** Daniel T. Halperin, Shira Doron, Stephen Hodgins, Robert C. Bailey, Stefan Baral, Rajiv Bhatia, Jeanne Noble, Monica Gandhi, Norman Hearst

**Affiliations:** 1 Department of Health Behavior, University of North Carolina, Chapel Hill School of Public Health, Chapel Hill, NC, USA; 2 Department of Medicine, Division of Infectious Diseases, Tufts Medical Center, Boston, MA, USA; 3 School of Public Health, University of Alberta, Edmonton, AB, Canada; 4 Division of Epidemiology and Biostatistics, University of Illinois Chicago School of Public Health, Chicago, IL, USA; 5 Department of Epidemiology, Johns Hopkins School of Public Health, Baltimore, MD, USA; 6 Department of Primary Care and Population Health, Stanford University School of Medicine, Palo Alto, CA, USA; 7 Department of Emergency Medicine, University of California San Francisco, San Francisco, CA, USA; 8 Division of HIV, Infectious Diseases and Global Medicine, University of California San Francisco, San Francisco, CA, USA; 9 Department of Family and Community Medicine, University of California San Francisco, San Francisco, CA, USA

## Abstract

The use of face masks has been widely promoted and at times mandated to prevent coronavirus disease 2019 (COVID-19). The 2023 publication of an updated Cochrane review on mask effectiveness for respiratory viruses as well as the unfolding epidemiology of COVID-19 underscore the need for an unbiased assessment of the current scientific evidence. It appears that the widespread promotion, adoption, and mandating of masking for COVID-19 were based not primarily on the strength of evidence for effectiveness but more on the imperative of decision-makers to act in the face of a novel public health emergency, with seemingly few good alternatives. Randomized clinical trials of masking for prevention of COVID-19 and other respiratory viruses have so far shown no evidence of benefit (with the possible exception of continuous use of N95 respirators by hospital workers). Observational studies provide lower-quality evidence and do not convincingly demonstrate benefit from masking or mask mandates. Unless robust new evidence emerges showing the effectiveness of masks in reducing infection or transmission risks in either trials or real-world conditions, mandates are not warranted for future epidemics of respiratory viral infections.

## Introduction

The coronavirus disease 2019 (COVID-19) pandemic and the response to it have caused great suffering and millions of deaths, upending societies, and disrupting healthcare systems around the world. Widespread immunity achieved through near-universal infection along with extensive vaccination, as well as the less virulent nature of the newer variants in circulation, has greatly reduced the burden of severe disease and death but clearly has not stopped transmission. Multiple community-level interventions, including home quarantines, school and business closures, social distancing, and mandatory masking, ultimately proved unsuccessful at containing the virus.^1^ Indeed, COVID-19 does not have the features of an eradicable disease because: (1) it is present in at least 29 animal species, (2) it can spread at the pre-symptomatic stage, (3) it has symptoms indistinguishable from those produced by other respiratory pathogens, and (4) it can still infect people following previous infection or vaccination.^2^

Over 4 years since its original appearance, COVID-19 has become endemic throughout the world, constituting a far smaller contributor to morbidity and mortality in the general population.^3^ The disease is not overwhelming healthcare systems, and the public health emergency is over. The main remaining questions are how to manage severe acute respiratory coronavirus virus 2 as an endemic virus and how best to prepare for potential future respiratory pandemics.^1,2^

Face masks have been one of the most widely promoted measures for reducing the spread of COVID-19. Indeed, early in the pandemic, several of the authors of this article were vigorous proponents of masking.^4^ Masks have been mandated or recommended around the world over the past 4 years and are still required today in certain settings, with some experts and authorities continuing to urge mandating their use as the virus periodically spikes in various regions. But face masks remain controversial. Intuitively, it may seem that wearing masks *should* work, but actual evidence on how well they work to reduce infection or transmission in real-world settings is limited, and results have generally been disappointing when evaluated in rigorous trials. Although for the past several years there has been wide consensus that masking outdoors is generally unnecessary,^1,5^ many public health experts and institutions continue to assert that masking indoors is a “proven” effective prevention method.^6^

A 2023 Cochrane analysis examined the extant evidence on the effectiveness of face masks and other physical measures to prevent the spread of respiratory viruses.^7^ This review updated an earlier Cochrane assessment completed before the COVID-19 pandemic and included studies specifically regarding the effectiveness of masks against the coronavirus. This recent analysis along with ongoing shifts in the dynamics of COVID-19 offers an opportune moment to reevaluate the role of face masks in mitigating this now endemic disease and in preparing to deal with the next epidemic respiratory virus.

## The past

Before COVID-19, face masks and/or respirators like N95s were required in certain clinical contexts, including use of masks for sterile procedures such as surgery or respirators when caring for patients infected with certain known or suspected respiratory pathogens (eg, tuberculosis) or having seriously compromised immune function. There was no general requirement for masking among healthcare workers, patients, or visitors to health facilities.

Outside of healthcare settings, there were no mask requirements. Some people, particularly in several East Asian countries, chose to wear masks during the winter influenza season or when they had cold-like symptoms, or during periods of pollution-driven poor air quality. Doctors recommended face masks for patients in specific situations, such as neutropenic patients going into crowded places during influenza outbreaks.

After the arrival of COVID-19, masking was quickly adopted around the world (with a few exceptions, such as in Sweden^5^). The push to do so appeared irresistible and was not simply a matter of following the lead of organizations such as the World Health Organization and the Centers for Disease Control and Prevention (CDC). Among decision-makers, there appeared to be an imperative to do *something* in response to a major new public health threat; with few good options available, people wanted to feel protected.^8^ Once the ball got rolling, officials of all types, including health experts, politicians, college presidents, school board members, and business leaders, started ordering masking in almost all settings.

Such decisions may have made sense to leaders who saw little downside to ordering masking and who risked criticism if they did not. But the sudden decision in early 2020 by agencies such as the CDC to embrace masking appears not to have been based on any new evidence of effectiveness. (In fact, some experts early on cautioned that tiny coronavirus aerosol microparticles are largely unhindered by face coverings.^9^) Mask mandates were often introduced along with or as the preferred alternative to stay-at-home orders, lockdowns, and school closures. Extended lockdowns were untenable, yet it was much easier to maintain mask requirements over the longer term; some have even been continued to the present day, particularly in healthcare settings.

In retrospect, there may have been a rationale for imposing mask mandates at a time when the death toll surged and hospitals were threatened with becoming overwhelmed. Even a small decrease in infections, had it been achieved, could have been worthwhile. (Furthermore, a theory was proposed regarding the potential for masks to decrease the size of the inoculum and thus severity of illness.^10^) Perhaps the main problem was not the decision to implement mask mandates initially but the certainty with which their effectiveness was communicated to the public, the failure to immediately and rigorously assess the effects of those mandates, and prolonging them in the absence of discernible effect. In any case, our objective is not to criticize those who made such decisions but to understand how we got to where we are and what decisions should be made (or not) in the future.

## The evidence

Where do Cochrane reviews fit in? Cochrane is an international nonprofit organization that supports the development of rigorous systematic reviews on important health-related topics. Cochrane does not conduct new research itself but provides a formalized process for gathering, synthesizing, and assessing existing research findings. Cochrane reviews follow a standardized methodology and are subject to careful evaluation by the organization beyond the usual scientific peer review process.^11^ Cochrane reviews have often been considered the gold standard for resolving controversies in medicine,^12^ especially when individual studies have shown inconclusive or conflicting results.

Cochrane evidence synthesis primarily draws on the findings of randomized controlled trials (RCTs), widely agreed to represent the strongest level of evidence for assessing intervention effectiveness. Studies lacking randomization into intervention and comparison groups are considered lower-quality evidence because they are more vulnerable to various biases.^13^ The need for RCTs when evaluating drugs and other medical treatments has long been recognized, and this is also true for preventive interventions. As with most other Cochrane reviews, the January 2023 review of masking and other physical measures to prevent the spread of respiratory viruses therefore only considered data from RCTs in its analysis.

This latest review was the 6th in a series of Cochrane reviews assessing the effectiveness of face masks, going back to 2007. Although the 2023 review included several new studies conducted during the COVID-19 pandemic, incorporating these new data did not change the conclusion of the previous review: the combined evidence of RCTs examining face masks to prevent viral respiratory infections still had not demonstrated any measurable benefit. There was no convincing evidence in the latest report to suggest that face masks work any better for COVID-19 than they do for other respiratory viruses.

Perhaps the most striking finding of the review is that, despite COVID-19 rapidly surpassing HIV as the most researched virus in history,^14^ the world literature included only 2 RCTs conducted in the COVID era comparing face masks to no mask. A study in Denmark found that surgical masks made no significant difference on the risk of acquiring COVID-19.^15^ A large community study conducted in Bangladesh found a slight protective effect for an intervention intensively promoting use of surgical masks, but no impact for cotton masks.^16^ This was seen mostly in the oldest age group and was of borderline statistical significance.^17^ A subsequent reanalysis of the data found that even this borderline finding may have been due to bias caused by irregularities in the randomization process.^18^ A third RCT investigating mask use to prevent COVID-19 was published after the latest Cochrane review was released. This similarly large community trial conducted in Guinea Bissau found that providing free face masks and messages about correct use had limited impact on either mask use or morbidity and mortality.^19^

Despite the prominence given to masking requirements and recommendations over the past 4 years, nowhere near as much effort has been devoted to rigorous assessment of the impact of this core intervention. Almost no research has been forthcoming from organizations including the National Institutes of Health and the CDC, nor have they issued requests for proposals to support external research on this vital question which has affected so many people’s lives. Conducting RCTs to test face masks is not easy, but the 17 studies included in the Cochrane review show that it is possible.

Why has more research not been done? For one thing, it appears that once public health officials and policymakers became committed to this strategy, there was strong resistance to any questioning of mask effectiveness.^5,14,20^ To defend mandates, authorities characterized the value of face masks (or often of any face covering) as an established scientific fact. In some settings, conformity to this position became an acid test of public health commitment.^2,14,21^ There were instances of experts being censored on social media for questioning the effectiveness of masks, and California passed legislation (which was repealed a year later) that would enable punishment of physicians who discouraged or questioned mask use.^22^ Conducting research to evaluate the effectiveness of face coverings might have called the strategy into question. The sad result is that, 4 years later, we have learned far less about mask effectiveness than we could have.

The latest Cochrane review provided new pooled estimates of the effectiveness of masking. Meta-analysis involves mathematically combining the findings of RCTs conducted in different populations—often with somewhat different methods—but that poses a similar question: in this case, whether face masks offer protection against viral respiratory infections. Although some masks may work better in some situations than others, the overall pooled estimate combining all RCTs that meet strict inclusion criteria is the widely accepted way to examine if there is any class effect.^23^ If masks were anywhere near as effective as is widely assumed, this methodology presumably should have been able to detect some level of effect from such a combined analysis. If there were a class effect, it then would be reasonable to go back to see which types of interventions seemed to work best. For the 12 RCTs reviewed in the latest Cochrane report that compared cloth or surgical masks to no masking, the combined estimate found no evidence of a protective effect.

For RCTs comparing respirators to simpler cloth or surgical masks, the results were less straightforward. The combined Cochrane analysis of 5 RCTs (4 of them conducted in healthcare settings) found a trend favoring N95s, however this did not reach statistical significance. Two very similar RCTs^24,25^ from China in which hospital workers continuously wore N95s appeared to show a benefit, and after pooling findings from these 2 studies into a combined analysis, the results reached statistical significance.^26^ However, 2 RCTs conducted among healthcare workers caring for symptomatic patients in the United States^27^ and in Canada^28^, comparing targeted use of N95s to surgical masks, showed no impact. (There have been no RCTs published to date comparing the use of N95s to no mask use.) The discrepancy in such findings may be in part due to consistency of use; unlike other health interventions such as condoms, all respiratory devices need to be worn continuously and correctly, often for many hours at a stretch. This limiting factor is even more crucial given that the bulk of transmission has occurred in household settings among family, friends, and roommates^5,29^ (and of course, such settings were not—and could not realistically be—covered by mandates).

Many public health experts and agencies, rather than relying on the RCT evidence, have affirmed the effectiveness of masking based on the results from observational studies. There have been many observational studies of various types, and the findings of some, indeed, would appear to suggest that masks are effective. Such studies fall into 2 main categories. One involves comparing people who chose to use masks to those who did not, thus involving various self-selection biases. For example, a widely cited CDC case-controlled (non-peer-reviewed) study of people attending COVID-19 testing sites^30^ found that those testing negative reported more face mask use than those testing positive. But instead of proving that masks work (as was widely announced), this finding may very well have resulted from habitual mask users being more COVID-conscious in general, more likely to seek testing, and therefore more likely to test negative on any given occasion.^31^ (The CDC survey’s methodology was further weakened by an extremely low response rate of around 10%.)

The other important category includes ecological studies that compare 1 or 2 counties or school districts that enacted mask mandates to others without such mandates and thereby attribute differences in COVID-19 rates to the mask mandates.^32^ Such comparisons have also been widely cited by health authorities and even published in leading medical journals as evidence that masks work.^33^ But other studies that included a larger number of geographic units (such as all California counties^34^ or across the entire country of Switzerland^35^) have shown no association between mask mandates and COVID-19 case rates. Even ecological studies with more geographic units are subject to bias. One such study claimed to show much lower in-school transmission of COVID-19 in schools with mask mandates.^36^ But the schools followed CDC criteria that counted cases as “in-school” only if there had been a known mask-less exposure. The schools with mask mandates consequently had fewer cases classified as “in-school” but more cases classified as “community acquired.”^37^

One ecological argument often invoked in support of mask effectiveness is that mask use was more common in East Asia, and some of those countries were indeed relatively successful in controlling COVID-19, at least early on. China, in particular, was able to hold COVID-19 in check for nearly 3 years with a strategy that included widespread mandating of mask use. Although masks may have helped, it is impossible to know; the Chinese strategy also included frequent mass testing, forced isolation in centralized facilities of people testing positive, contact tracing and quarantine of people potentially exposed, transportation restrictions, and intermittent hard lockdowns confining millions of people to their homes for weeks at a time or longer, making it impossible to tease apart the specific contributions of any of these various measures. Moreover, in December 2022, COVID-19 quickly exploded in China after these stronger measures were relaxed, even though masking rules continued. Hong Kong, which has had the strictest mask mandates in the world and where the use of surgical masks and N95s is ubiquitous, had the world’s highest COVID-19 death rate during parts of 2022^38^ and 2023.^39^

Such examples demonstrate the pitfalls of relying on observational or ecological data to determine if an intervention such as face masks is effective. Even if the intervention does not work, inevitably some observational studies will appear to show that it does. Even large apparent benefits may be mainly or entirely due to factors other than the actual intervention. Because of their inherent vulnerability to selection and other biases, even well-designed observational studies constitute relatively weak evidence to investigate direct causal effects. This is the important advantage of the Cochrane approach of identifying and assessing all high-quality RCT data, using a standardized protocol that precludes cherry-picking of studies. If any other medical intervention had been found to have no significant effect in a Cochrane synthesis of 12 RCTs (as has now been performed for cloth and surgical masks), presumably few experts would argue for the necessity to examine the observational data instead.

## The bottom line

Over 4 years into COVID-19, we still have no conclusive evidence to justify requirements or even strong recommendations for the use of masking for the prevention of COVID-19 or other respiratory infections in community settings. RCTs have been few in number and have not demonstrated benefit (except possibly for N95s in certain clinical settings), and as discussed, the widely cited observational studies offer weak evidence for a beneficial effect of mask mandates on COVID-19 rates.

Does this mean masks are inherently ineffective? Not necessarily. One criticism of the RCT evidence is that you cannot get enough subjects in the mask-assigned groups to actually use them consistently and correctly.^40^ So, do mask trials show no effect because masks are ineffective? Or do they show no effect because people do not use masks enough, or use them correctly enough, even when urged to do so? The practical result either way is that mask *mandates*, as a public health strategy, appear to be ineffective. Mandates that do not work cannot be justified.^1,2^

It is theoretically possible that mask use may have some protective effect that is missed in all the noise of research data or that respirators, particularly, if used correctly and consistently by motivated individuals at high risk, may provide meaningful protection. However, even if so, we still have no idea of how much protection they may offer or what degree of use is necessary to achieve substantial protection. These are researchable questions that need to be investigated if masks are to be retained as part of our prevention toolbox in the post-vaccine world or if we are to consider masking again for future epidemics of other respiratory viruses.

COVID-19 is now endemic. Fortunately, the frequency of severe illness among those who are vaccinated and/or have immunity from prior infection has fallen to where it is now of a similar order of magnitude to the usual risks posed by other respiratory viruses, such as seasonal influenza. The additional trials included in the latest Cochrane review provided no evidence that masks work better to prevent COVID-19 than they do to prevent infections from other respiratory viruses. Mask mandates have been divisive, and robust evidence that they reduce risk of transmission is lacking. Obviously, interventions with theoretical or experimental value still require real-world evaluation. The history of medicine is full of examples of interventions that intuitively should have worked, based on theory or laboratory-based evidence, but turned out not to work in practice.

## Recommendations

The only circumstance in which RCTs possibly suggest a benefit from policies or programs utilizing masks or respirators is when well-fitted N95s are worn correctly and continuously in specific clinical settings. It is a big stretch, based on this potentially positive evidence alone, to conclude that large-scale, community-level mandates could ever produce similar impact. It therefore follows that masking should be deemphasized as a general population strategy for preventing COVID-19 or transmission of other respiratory viruses, unless or until better evidence for benefit accrues.

Of course, there should be no objection to anyone who chooses to wear a mask or respirator. The healthcare system should help such people to mask in a way most likely to produce benefit, which most likely involves consistent use of a well-fitted N95. Any recommendation to use masks should be targeted to individuals and circumstances involving high risk. Yet such recommendations should not be provided along with unwarranted assurances of certainty. We can inform people at high risk that masking may provide them some protection, but we must be honest, acknowledging that no one knows for sure what degree of protection, if any, this will offer. Studies performed on mannequins do not always generalize well to humans. If this means that some choose not to mask, that is also a reasonable choice. The still common practice of healthy people wearing masks to protect vulnerable family members may be commendably altruistic but also is not grounded in robust evidence.

Mandates, including mask mandates, are not ethical in the absence of proven effectiveness. Indeed, there is now fairly strong evidence that mask *mandates* are not effective. Furthermore, masks are not completely benign^5^; they result in communication barriers, especially for the hearing impaired^41^; psychosocial harms, particularly for young children^42,43^; and immense volumes of environmental waste.^44,45^ Even without mandates, the current practice of “highly recommending” masks in settings where almost no one still uses them further undermines the credibility of public health officials.^46^

Regarding healthcare settings, the dearth of research, and in particular the absence of studies comparing N95 or other respirator use to no mask use, makes it difficult to conclude one way or another whether masks, or more likely respirators, can prevent transmission of respiratory viruses. Mask mandates were not common prior to the COVID-19 pandemic, and COVID-19 no longer poses risks qualitatively or quantitatively greater than those of other diseases we have long faced. It no longer poses an unusual threat to healthcare workers. There is thus little justification to support universal masking in medical settings. Indeed, good communication and empathy, including noticing subtle nonverbal cues, are essential to high-quality medical care and are hindered by masking.^47^ We believe mask policies in medical settings should therefore revert to the usual practice prior to COVID-19.

Above all, we should drop all masking requirements and recommendations in schools. Children are the demographic least medically affected by COVID-19^1,5,48^ and most likely to be harmed by mask mandates.^49^ Initial fears that schools might be hotbeds of infection and drivers of community transmission have repeatedly been shown to be unfounded.^1,5,50^ Countries that never implemented masking in schools, such as Scandinavia, did not see higher rates of COVID-19 among students or staff and did not have worse COVID epidemics than other countries.^51^ Schools should be the last setting in which masks are required or recommended rather than the first. At the other end of the age spectrum, older people should not be told they need to wear masks, or to have those around them wear masks, for the duration of their lives.

Inevitably, there will be serious new outbreaks of respiratory viruses in the future. Based on current evidence, we can have little confidence that mask mandates will meaningfully contribute to their control. Indeed, we have a sobering lack of evidence that cloth and surgical masks would be ineffective for that purpose. Perhaps N-95 and similar respirators, correctly and consistently worn, would be somewhat helpful in certain situations. But strong evidence for their effectiveness in reducing transmission remains limited, compounded by the unshakeable reality that it is unlikely many people will adhere to proper and consistent use of respirators. Reflecting on our experience with the COVID-19 response, we can do better in the future by:

- Strengthening the evidence base—now—regarding the efficacy of interventions that may potentially reduce transmission risk (eg, more RCTs of N-95 respirators).
- Resisting the temptation to impose top-down measures when the evidence for their effectiveness is lacking.
- Being more honest in our public communications about the limitations of the evidence; one aspect of the duty of public health professionals is to acknowledge a level of uncertainty.
